# Mapping mammalian synaptic connectivity

**DOI:** 10.1007/s00018-013-1417-y

**Published:** 2013-07-18

**Authors:** Chaehyun Yook, Shaul Druckmann, Jinhyun Kim

**Affiliations:** 1 Center for Functional Connectomics (CFC), L7-7205, Korea Institute of Science and Technology (KIST), 39-1 Hawolgokdong, Seongbukgu, Seoul, 136-791 Korea; 2 Department of Biological Science, KAIST, Daejeon, Korea; 3 Howard Hugh Medical Institute, Janelia Farm Research Campus, Ashburn, USA

**Keywords:** Synaptic connectivity, Brainbow, Array tomography, mGRASP, Trans-synaptic tracer, Neurological disorders

## Abstract

Mapping mammalian synaptic connectivity has long been an important goal of neuroscientists since it is considered crucial for explaining human perception and behavior. Yet, despite enormous efforts, the overwhelming complexity of the neural circuitry and the lack of appropriate techniques to unravel it have limited the success of efforts to map connectivity. However, recent technological advances designed to overcome the limitations of conventional methods for connectivity mapping may bring about a turning point. Here, we address the promises and pitfalls of these new mapping technologies.

## Introduction

More than a century ago, the visionary Spanish neuroanatomist Santiago Ramón Cajal (Nobel Laureate 1906) proposed what is often called the “neuron-doctrine,” the idea that neurons are the structural and functional units of the brain, and guided by this idea, proceeded to explore the complex architecture of neuronal networks [1, 2]. Neurons in networks communicate with one another through a special bridge-like structure called a synapse. Neuronal connections were traditionally determined by electrophysiological measurement from linked pairs of cells to determine the strength as well as the existence of a synapse, yet this approach has a very low throughput [3]. To this day, neuroscientists continue to seek new high-throughput ways to investigate neuronal circuits by mapping synaptic connectivity [4–10]. Recently, in 2005, the terms “connectome” and “connectomics” were coined and have since been widely used to describe this effort [11] (“-ome” as an analogy to “genome” is taken to signify complete maps of connections in a brain or a brain area). In 2009, the Human Connectome Project was launched by the National Institutes of Health (http://www.humanconnectomeproject.org) with the goal of building macro-scale descriptions of structural and functional connectivity in healthy human brains (strictly speaking, it would have been better named the “projectome” since the methods used in the Human Connectome Project can provide only macro-level profiles of nerve bundle projections); these profiles can be used to predict the probability of connectivity but not specific connections between given cells [9, 12, 13], and, increasingly, scientific endeavors are underway to map synaptic connectivity in other organisms (e.g., nematode, fruit fly, and mouse) at multiple scales: micro-scale (nano- or micrometer resolution) for synapse-by-synapse or neuron-by-neuron connectivity, meso-scale for local circuits, and macro-scale (millimeter resolution) for entire brains [4, 14, 15].

However, there are fundamental challenges to reconstructing synaptic connections. First, the synapse is a nanometer-scale structure located along neuronal processes that are very fine (less than a micrometer in diameter), but also very long (sometimes more than a few millimeters in length). Accordingly, synaptic reconstruction requires both high-resolution and large-scale mapping, which are difficult to achieve together. Second, in many areas, neurons with interdigitating processes are packed very densely in networks, so that disambiguating individual neurons is time- and labor-intensive. Given these challenges, as was also the case for the Human Genome Project, skeptical views on the connectomics project have emerged (the great brain mapping debate at Columbia University, 2012) [16]. A cautionary perspective is provided by the only organism whose entire connectome and genome have been mapped, the nematode *C. elegans*, which has 302 neurons and 97 megabases (compare with 86–100 billion neurons and 300 megabases in the human) [14, 17]. Some neuroscientists point out that we still do not understand how some of the most basic behaviors of this tiny creature are governed despite its simplicity, and suggest that systematic connectivity mapping will not provide efficient ways to answer questions about brain function.

Yet, most neuroscientists agree that furthering knowledge of synaptic connections and extracting the principles of these connections will aid in understanding how the brain works [8, 18, 19]. Some skeptics focus on the cost-gain economics of the enterprise since the capacity of currently available techniques for connectivity mapping indeed forms a bottleneck. Therefore, developing and improving new technologies for synaptic mapping will be essential at each step of the process: neuronal labeling, imaging, and reconstruction. In Cajal’s time, the valuable staining method developed by Camillo Golgi (Nobel Laureate 1906) in 1873, an up-to-date Zeiss microscope, and his talent for drawing allowed him to discover many important aspects of the organization of the nervous system [1, 2, 20, 21]. In modern times, the discovery of fluorescent tags, advanced genetic engineering for labeling, innovations in optics for imaging, and tremendous increases in computer power for reconstructing all facilitate connectomics. Here, we review advanced techniques for mapping connectivity, their promise and pitfalls, with particular attention to visualizing connectivity.

I. Electron-based imaging approach
II. Photon-based imaging approach

II-1. Brainbow

II-2. Array Tomography

II-3. mGRASP

II-4. Trans-synaptic tracing

II-5. New LM: Super-resolution LM and SPIM with transparent brain

III. Functional connectivity
IV. Synaptic connections in neurological disorders
V. Interactions with computational modeling

## Electron-based imaging approach

**Fig. 1:**
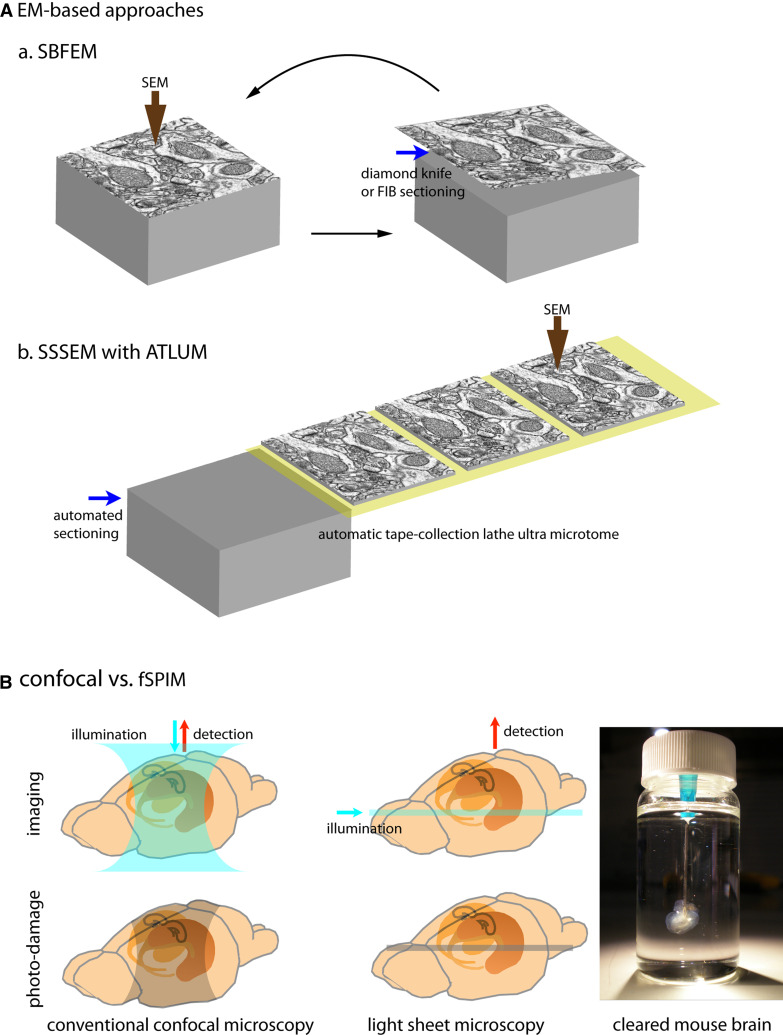
EM approaches and new LM (fSPIM)

Electron microscopy (EM) developed by the German physicist Ernst Ruska (Nobel Laureate 1986) in 1931 provides much better lateral resolution (approximately 50-pm resolution) than light microscopy (LM), which is limited by diffraction (~200-nm resolution). Crucially, resolving the ~20-nm-wide synaptic cleft of a densely packed synapse is beyond the resolution of typical LM but is well within that of EM. Accordingly, EM-based approaches have been considered the option of choice for complete synaptic reconstruction. While a sparse-mapping approach allowed Cajal to propose his connection diagrams using information only about a few Golgi-labeled neurons at a time, EM-based dense-mapping now provides a relatively complete picture of neuronal structure [14]. EM dense reconstruction allows achievements that are almost impossible to achieve by LM-based mapping, yet the process of synaptic mapping through EM, especially data acquisition and reconstruction, is extremely time- and labor-intensive. Therefore, much effort has been devoted to improving the throughput of the EM process. To improve data acquisition, several new approaches have been developed (Fig. 1A). Serial block-face electron microscopy (SBFEM) is designed to obtain well-aligned images by serially imaging back-scattered electrons from the surface of tissue embedded in a sample block, then slicing that surface away, and then imaging the newly revealed surface. The sample block is sliced by either a diamond knife [22] or focused ion beam (FIB) [23] incorporated into the EM chamber. With a diamond knife, SBFEM has been reported to have voxel resolution of 20 × 20 × 25 nm [4, 24]. The lateral resolution of scanning electron microscopy depends on field-emitted electron density; its *z*-resolution can be improved by cutting thinner slices, and, in case of SBFEM, by using lower electron beam energy to limit penetration depth [22]. Serial section scanning electron microscopy (SSSEM) combined with a sample collection system (automatic tape-collection lathe ultra microtome, ATLUM) offers voxel resolution as high as 4 × 4 × 50 nm as well as reliable section collection with reduced section distortion [24]. Both systems have sufficient resolution to trace dense neuropils and to reconstruct the synaptic connectivity of neural circuits. For the resolution needed to observe even gap junctions, automated transmission EM (ATEM) offers a combination of higher-resolution serial TEM with automated image alignment and registration [25]. However, putting aside that EM-based imaging processes take relatively more time than LM-based processes, it is the analysis of these images and the reconstruction of neurons and their connections that really hold back the progress of EM-based connectivity mapping. Since an EM image contains every visible detail within its field of view, selecting relevant information, such as distinguishing contours of interest from irrelevant ones, takes much more time than would be the case for a sparse-labeled LM image (200–400 h/mm for manually tracing SBFSEM images versus 0.25–1 h/mm for single neuron-labeled LM images) [24, 26, 27]. To expedite this intensively time-consuming step, efforts to develop a reliable computer-based tracing method, such as through machine-learning algorithms, are underway [28, 29]. However, thus far, unfortunately, even after important new advances in EM-based methodology, reconstructing neuronal tissue remains a relatively time-consuming and volume-limited endeavor.

## Photon-based imaging approaches

**Fig. 2:**
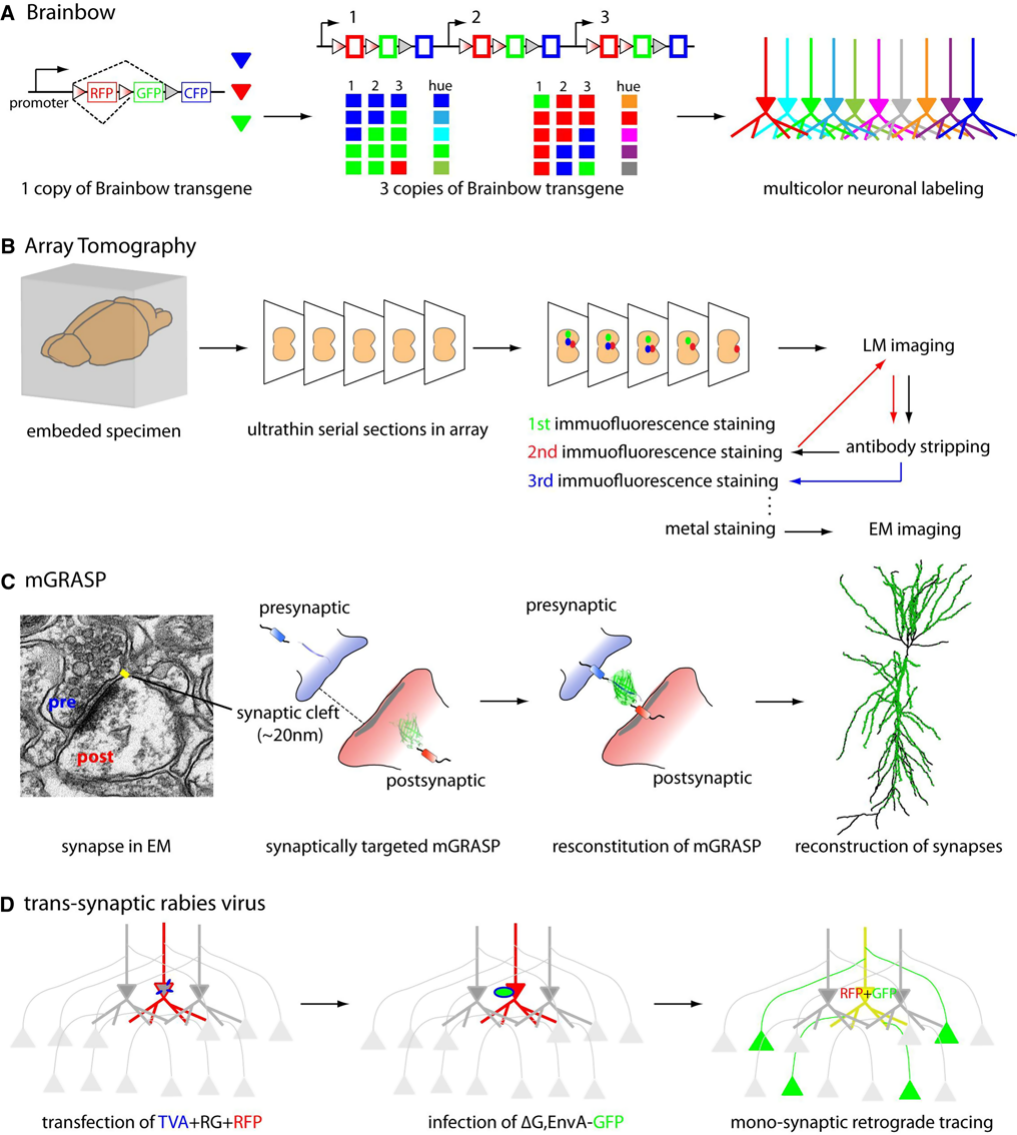
Principal of LM approaches for mapping mammalian synaptic connectivity

Recently, more sophisticated genetic and optical methods have been advanced to circumvent the low resolution of photon-based strategies for mapping synaptic connectivity (Fig. 2).

### Brainbow

Historically, the degree of overlap between the axonal arbor and the dendritic arbor of sparsely labeled neurons, as measured by LM, has been used to infer the presence of synaptic connectivity [8]. However, unless the labeling is very sparse, it is difficult to reconstruct the processes of individual neurons. To disambiguate each neural process from densely packed axons and dendrites, Lichtman and his group [7, 30, 31] set out to label thousands of neurons with random colors drawn from a set originally consisting of membrane-permeable fluorescent dyes, and later of fluorescent proteins. Genetic labeling can be achieved through an elegant approach using genetic switches and multi-copied transgene integration, called Brainbow [31]. Brainbow is based on the combinatorial and stochastic expression of concatenated multiple fluorescent proteins (collectively referred to as XFPs), in which each spectrally distinct XFP is floxed with variants of the loxP site. Multiple copies of this transgene are inserted into the mouse genome. Stochastic recombination events mediated by Cre recombinase determine which combination of XFPs from the multiple copies of transgene will be formed. The many possible combinations of XFP transgenes result in many unique colors for different neurons. For example, if there are three transgene copies in a Brainbow mouse line, each containing three spectrally distinct XFPs, a neuron can have one of ten hues. Neurons labeled with different colors can be distinguished from one another much more easily. Accordingly, by separating the different color channels, Brainbow allows neurons to be reconstructed one-by-one even within a dense population, thereby extending information obtained by LM-based tracing to the level of the neuronal population. In other words, Brainbow permits the challenging dense reconstruction problem to be addressed by solving the much simpler sparse neuron reconstruction problem. In addition, Brainbow greatly simplifies projection pattern reconstruction since the unique color of each neuron remains recognizable along the projection, and therefore usually there is no need to trace the projection all along its path. This results in significant savings in time when tracing long-range circuits. Lastly, Brainbow can assist time-lapse imaging or developmental studies since the unique color profile of each neuron remains stable. Very recently, a new second generation of Brainbow has been developed that uses better detectable and antigenically distinct fluorescent proteins or flp recombinase-FRT (Flpbow) [32]. On the negative side, although Brainbow allows identification of neuronal processes by differentially coloring individual neurons, and allows their synapses to be inferred from neurite contacts, the diffraction-limited resolution of LM is insufficient to unambiguously confirm the presence of actual synapses. This inference technique thus appears to be useful for only a subset of synaptic connections [33, 34]. Thus, additional technologies are usually needed to identify synapses.

### Array tomography

Array tomography (AT) combines LM and EM approaches to resolve synapses by using multiple antibodies to label synaptic markers [35, 36]. It benefits from the high throughput of LM, high *z*-resolution of EM, and improved quantitative reliability of information obtained through multi-immunofluorescence. To use this method, a mouse brain is embedded in hydrophilic resin and is then serially sectioned into 50–200-nm ultrathin slices. Then, a long array of these serial sections is repeatedly stained for immunofluorescence by a large number of multiplex markers. Because samples prepared for AT are also compatible with EM, AT can combine LM and EM images. Ultrathin physical sectioning overcomes some obstacles typically presented by immunofluorescence staining and imaging approaches: it permits depth-independent immunostaining and imaging and improves upon the *z*-axis resolution of conventional confocal microscopy (~700 nm). The protein components of individual synapses are revealed by large sets of markers labeled through repeated cycles of antibody stripping and re-staining. This process, called a single-synapse analysis or a synaptogram, offers insights into synapse molecular diversity [36]. In addition, automated steps for imaging and aligning serial sections allow the reconstruction of neural circuits in large volumes of tissue. Thus, AT reveals not only anatomical circuits but also the synaptic proteome. These combined benefits are a unique advantage. However, a potential disadvantage is that AT relies entirely on antibody staining, leading to concerns that some methods for preparing brain tissue can sharply decreases its antigenicity, and that the fidelity of costly antibodies can vary significantly, potentially resulting in ambiguous and incomplete results.

### mGRASP

Another new genetically controlled method to resolve synapses at the level of LM, termed GFP reconstitution across synaptic partners (GRASP), is synapse-specific labeling with two complementary GFP components [37, 38]. GRASP is based on two non-fluorescent split-GFP fragments (called spGFP1-10 and spGFP11) tethered to synaptic membranes in each of two neuronal populations. When two neurons, each expressing one of the fragments, are tightly opposed across a synaptic cleft, fluorescent GFP is reconstituted. More recent mammalian GRASP (mGRASP) techniques can precisely label actual synapses, not non-synaptic membrane contacts, by engineering spGFP carriers that are specifically targeted to synaptic membranes and that accommodate the physical spacing of the synaptic cleft [38]. The manifest benefit of mGRASP technology is the accurate nanometer-scale (~20 nm) detection of synapses while circumventing the diffraction limitations of LM. When tested with known synaptic and non-synaptic connections in samples full of axonal contacts, mGRASP has been shown to specifically detect actual synapses with no or few false positives. When combined with specialized analysis software [38, 39], mGRASP can relatively quickly reveal the precise locations and numbers of synapses along postsynaptic dendrites, sites responsible for determining many important characteristics of signal processing. Thus, mGRASP technology is suitable for imaging large-scale connectivity patterns. A potential concern, though, is that this technique sometimes registers false negatives, making it difficult to determine absolute numbers of synapses. The problem of false negatives is common to all LM approaches and varies with instrumental parameters (i.e., laser power, emission spectra, etc.). Further optimization of mGRASP technology and applying it in combination with other technologies will lead to useful new tools for mapping mammalian synaptic connectivity.

### Trans-synaptic tracing by rabies virus

Another method using LM is based on the anterograde and retrograde label of neuronal circuits by trans-synaptic tracers. Among retrograde viral trans-synaptic tracers, the modified rabies virus system seems to be the most promising for mapping synaptic connectivity [40–42]. The rabies virus is a negative-sense, single-stranded RNA virus enveloped by rabies glycoprotein (RG) [43, 44] that travels retrogradely between synaptically connected neurons. Interactions between the RG and its receptor in host cells are crucial for the initial and the subsequent trans-synaptic infections. The receptor for RG appears to be restricted to presynaptic nerve terminals [45], possibly explaining the highly selective retrograde trans-synaptic spread of rabies [46–48]. The synapse specificity of rabies virus spread has been assessed anatomically and electrophysiologically in the context of well-characterized neuronal connections [49, 50]. Wild-type rabies may make multi-synaptic jumps, making it difficult to determine which of the labeled neurons is directly connected to which other neuron. However, recently developed genetic modifications, such as the deletion of an essential envelope glycoprotein (∆G) that is trans-complemented in only specific cells, allows mono-synaptic retrograde tracing [41, 42, 51]. Specifically, ∆G-rabies virus packaged with the avian virus envelope protein EnvA, will only infect cells expressing avian virus receptor TVA, one not expressed in mammalian cells [52]. Delivery of TVA along with RG into a specific set of neurons followed by ∆G-rabies virus expressing GFP, for example, allows visualizing synaptically connected pairs of neurons. Specific expression of TVA and RG is achieved by using cell-specific Cre mouse lines or by single-cell electroporation in vivo. Further engineering of the rabies virus-based system, for instance, to generate a modified version with lymphocytic choriomeningitis virus (LCMV) glycoprotein, will enable anterograde monosynaptic tracing, offering directional choice [53]. This method is promising and powerful not only for investigating synaptic connectivity and performing physiological characterizations, but, when implemented with recombinant viruses expressing activity indicators (e.g., GCaMP), can also be used to monitor neuronal activity in specific circuits [40]. To date, however, these genetic products are sometimes fairly toxic, and trans-synaptic jumps can be biased by cell type leading to incomplete (40–85 %) presynaptic labeling [49], thus limiting the utility of this technique.

### New LM: Super-resolution LM and SPIM with transparent brain

Thus far, we have described methods for overcoming the diffraction limit of LM to resolve synapses by genetic multiple coloring (Brainbow), synaptic immunolabeling with multiple markers (AT), and molecular engineering (mGRASP and rabies virus). Recently, super-resolution microscopy based on non-linear optics with nanometer resolution (i.e., SIM [54], PALM [55], STORM [56], STED [57]) has shown the potential to overcome the resolution limit of light. Such direct optical advances are helpful when combined with methods for mapping synaptic connectivity such as Brainbow and AT, but there is still room for improvement in mapping circuits in the large three-dimensional volume of the brain.

For the 3D reconstruction of an intact brain region, a whole mouse brain, or even a living creature, fluorescent selective plane illumination microscopy (fSPIM) along with optically cleared specimens have recently been introduced [58–60]. SPIM uses a thin laminar sheet of light to illuminate the focal plane, unlike confocal microscopy, which uses cone-shape illumination through the specimen and a spatial pinhole to remove out-of-focus rays. In fSPIM, only the focal plane is illuminated, greatly reducing depth-dependent photobleaching (Fig. 1B). In addition, providing illumination orthogonal to the detection axis allows for deep penetration of light independent of the depth of the focal plane for optical sectioning. This technique also alleviates section distortion and eliminates the challenges of image alignment. Very recent improvements to fSPIM include reducing the width of the light sheet and allowing multi-view reconstruction for better resolution and minimal scattering [61, 62]. Since fSPIM is most effective when used with transparent specimens, new optical clearing methods for fluorescence labeled tissues have been developed in parallel [59, 63, 64]. In particular, the recently developed clearing method called CLARITY improves a specimen’s imageable depth, and provides further improvements in transparency, the faithful preservation of fluorescent signals, and stabilization of the sample volume [64]. In our view, fSPIM and new clearing methods together with advanced synaptic detection methods (such as mGRASP and trans-synaptic tracers) are very promising developments for the complete mapping of mammalian synaptic connectivity.

## Functional connectivity

It is of critical importance to understand the relationship between the structure of a neuronal network and its function. Both the EM and LM approaches for visualizing connectivity described above provide only structural information about synaptic connectivity. In recent years, optogenetic approaches (e.g., channelrhodopsin) have accelerated mapping the spatial distribution of synaptic connections together with measures of synaptic strength [65, 66], yet these techniques can yield ambiguous results owing to the low resolution of opto-stimulation. To overcome this issue, recent studies have focused on a combination of optogenetic approaches and two-photon microscopic calcium imaging that can precisely detect active synapses innervated by different inputs [67, 68]. At the network level, a powerful, albeit technically challenging, way to determine functional connectivity is to first employ calcium imaging to characterize functional properties, then prepare and image the sample using EM to reconstruct the circuit [69, 70]. One such study demonstrated that a property of connectivity, asymmetry of wiring, contributes to a specific computation—direction selectivity: the dendrites of mouse starburst amacrine cells make highly specific synapses onto direction-selective ganglion cells in ways that depend on the ganglion cell’s preferred direction [70]. The relatively low throughput of the EM approach, though, hinders mapping structure and function in large volumes of whole circuits in the mammalian brain. Combinations of LM approaches described above with task-specific optimizations might also be suitable and may make these analyses more practical.

## Synaptic connections in neurological disorders

Although several neurological disorders may be caused by abnormal synaptic connectivity, efforts to understand this mechanism have been impeded by the lack of suitable techniques. Particularly, autism spectrum disorders (ASD) have been suspected to result from surfeits or deficits of synaptic connectivity [71]. ASD is a group of conditions characterized by impaired social interaction and communication, and restrictive and repetitive behaviors. The number of children diagnosed with ASD continues to rise and ADS has become an important social concern. Studies of genome-wide screening of ASD patients point to possible susceptibility genes [72–79], and several studies with genetically manipulated mice are presently investigating the consequences of losing these genes. Intriguingly, studies in these ASD model mice have reported possible alterations in synaptic connectivity in certain brain regions implicated in ASD. Shank3 mutant mice [80] showed decreased spine density in medium spiny neurons (MSNs) of the striatum, while eIF4E-overexpressing transgenic mice [81] showed increased spine density in pyramidal neurons of the medial prefrontal cortex (mPFC). Although these results are consistent with abnormal connectivity, direct evidence for this is lacking, especially with respect to lengthy connections among brain regions. mGRASP technology would appear to offer a useful approach to test ideas about ASD because mGRASP can quickly and accurately detect actual synapses. A clear understanding of connectivity characteristics associated with ASD will guide us to understanding its cause, and to better diagnosis and treatment.

## Interactions with computational modeling

Not only is synaptic reconstruction technically challenging, but analyzing the reconstructed connections is non-trivial as well. Unlike an organism’s genome, which is well-defined and relatively stable, connectivity is well known to be variable and dynamic, changing with, for example, experience-related plasticity. Nonetheless, powerful new computational analyses, coupled with newly revealed synaptic connectivity, have the potential to yield significant insights regarding the principles of synaptic connectivity. First, at the most ambitious level, if the full set of connections between all neurons in a network can be mapped (and single unit dynamics are known) then the dynamics of the full circuit can, at least in principle, be calculated [18]. Such an achievement could yield great insight regarding the operation of the circuit, for example the ability to compute the output associated with each input (though this would require additionally knowing the sign and weight of each mapped connection). Of course, even partial synaptic reconstructions could help constrain the assumptions made when developing models of neural circuits. For instance, existence of highly connected clusters can have a significant effect on a circuit’s dynamics [82] and would be difficult to directly ascertain without larger-scale synaptic mapping. Second, the functions of an unidentified neuron could be inferred computationally from the functions of known neurons connected to it. For example, the types of sensory input a neuron processes may be inferred from its connections to identified receptors, or the stimulus preference of a neuron may be deduced from the biases of its input neurons [70]. Third, the architecture of a circuit may provide clues about its computational function. For instance, if a circuit is found to have widespread lateral inhibitory connections, it may be implementing a “winner-takes-all” single-option selection computation whereby the selection of a maximally excited feature is sharpened by the inhibition of the rest of the network [83]. Fourth, analysis of the connectivity may allow us to discover substructures within a given network, revealing an important level of order in the network [84, 85]. Finally, since computational neuroscientists had, until recently, only very few synaptic reconstructions to work from, it is very likely that the novel scope and nature of the data revealed by modern synaptic mapping will inspire new computational methods to reveal conserved properties across different connectomes, and to test the relation between structure and function. In our view, mapping synaptic connectivity is likely to reveal discrete and repeating “modules” within a circuit, providing a level of description between the single neuron micro-level to the macro-level of the circuit as a whole. Such a finding would be immensely important as we strive toward a deeper understanding of neural circuits.

**Table 1:** Summarized advantages and limitations of synaptic mapping methods

Approach	Methods	Advantages	Limitations
EM-based	SBFEM [22, 23], SSSEM with ATLUM [24], and ATEM [25]	High voxel resolution to reconstruct the synaptic connectivity of all kinds of cells in dense neural circuits Complete picture of neuronal structure	Extremely time- and labor-intensive Volume-limited
LM-based	Brainbow [8, 31]	Dense reconstruction of multi-colored neurons using LM Ease and high speed of projection-pattern reconstruction Time-lapse imaging or developmental studies	Ambiguous detection of actual synapse
	Array tomography [35, 36]	Improved z-resolution Reliable reconstruction and quantitative analysis of neural circuits in large volumes of tissue. EM compatibility Insights into single-synapse proteome	Decrease of antigenicity and fidelity of costly antibodies
	mGRASP [37, 38]	Accurate nanometer-scale detection of synapses with LM Quick and suitable for imaging large-scale connectivity patterns	Obscures absolute number of synapses due to false negatives Limited to pairs
	Trans-synaptic tracing by rabies virus [41, 51]	Elucidation of unknown mono-synaptic connectivity throughout the brain Combinational possibility with neuronal activity monitoring and manipulating (e.g., GCaMP, ChR2)	Cytotoxicity of rabies infection Relatively highly biased to false negatives

## Conclusions and perspectives

We have reviewed techniques currently available for imaging mammalian synaptic connectivity (Table 1). Unfortunately, thus far, none of these techniques is perfect. None, for example, assesses synaptic strength and efficacy. Innovative new technologies are still required. Meanwhile, creative combinations of all the above techniques, possibly including functional assessments, will go a long way toward allowing mapping of the brain. Stochastic multicolor labeling of Brainbow combined with mGRASP, for instance, could identify the presynaptic partners of a given neuron; it would require labeling each neuron and preparing dense reconstructions of synaptic connectivity under LM. EM combined with new versions of Brainbow-expressing antigenically distinct fluorescent proteins might expand dense reconstructions to encompass long-range connectivity [32]. Also, mGRASP combined with a new retrograde label virus [86, 87] system could help unlock the secrets of disynaptic circuits as well as monosynaptic pairs of cells, and the common drawback of all methods for anatomical synaptic mapping, a lack of information about synaptic activity and strength, can be overcome through combinations of techniques including existing activity indicators and optogenetic tools.
